# Small Airway Dysfunction Measured by Impulse Oscillometry and Fractional Exhaled Nitric Oxide Is Associated With Asthma Control in Children

**DOI:** 10.3389/fped.2022.877681

**Published:** 2022-06-17

**Authors:** Liang-Mei Lin, Yu-Jun Chang, Kuender D. Yang, Ching-Hsiung Lin, Jien-Wen Chien, Jun-Kai Kao, Ming-Sheng Lee, Tsay-I Chiang, Ching-Yuang Lin, Yi-Giien Tsai

**Affiliations:** ^1^Respiratory Therapy Section for Children, Changhua Christian Children’s Hospital, Changhua, Taiwan; ^2^Epidemiology and Biostatistics Center and Big Data Center, Changhua Christian Hospital, Changhua, Taiwan; ^3^Departments of Pediatrics, Mackay Memorial Hospital, Taipei City, Taiwan; ^4^Department of Microbiology and Immunology, National Defense Medical Center, Taipei City, Taiwan; ^5^Division of Chest Medicine, Department of Internal Medicine, Changhua Christian Hospital, Changhua, Taiwan; ^6^Institute of Genomics and Bioinformatics, National Chung Hsing University, Taichung, Taiwan; ^7^Ph.D. Program in Translational Medicine, National Chung Hsing University, Taichung, Taiwan; ^8^Department of Recreation and Holistic Wellness, MingDao University, Changhua, Taiwan; ^9^Department of Pediatrics, Changhua Christian Children’s Hospital, Changhua, Taiwan; ^10^Department of Post-Baccalaureate Medicine, College of Medicine, National Chung Hsing University, Taichung, Taiwan; ^11^Institute of Biomedical Sciences, National Chung Hsing University, Taichung, Taiwan; ^12^Frontier Molecular Medical Research Center in Children, Changhua Christian Children Hospital, Changhua, Taiwan; ^13^School of Medicine, Kaohsiung Medical University, Taichung, Taiwan; ^14^College of Nursing, Hungkuang University, Taichung, Taiwan; ^15^Division of Pediatric Nephrology, Children’s Hospital, China Medical University, Taichung, Taiwan; ^16^School of Medicine, Chung Shan Medical University, Taichung, Taiwan

**Keywords:** allergic asthma, impulse oscillometry, fractional exhaled nitric oxide, asthma control, pulmonary function

## Abstract

**Background:**

Impulse oscillometry (IOS) and fractional exhaled nitric oxide (FeNO) are sensitive and non-invasive methods to measure airway resistance and inflammation, although there are limited population-based studies using IOS and FeNO to predict asthma control.

**Objective:**

This study aimed to investigate the utility of IOS and FeNO for assessing childhood asthma control in terms of small airway dysfunction and airway inflammation.

**Methods:**

This prospective observational cohort study enrolled 5,018 school children (aged 6–12 years), including 560 asthmatic children and 140 normal participants. FeNO, spirometry, IOS, bronchial dilation test, total IgE, and childhood asthma control test (C-ACT) were measured. FeNO, IOS, spirometry, and C-ACT results were correlated with childhood asthma with and without control.

**Results:**

Uncontrolled asthmatic children had abnormal FeNO, IOS, and spirometric values compared with control subjects (*P* < 0.05). IOS parameters with R5, R5-R20, X5, Ax, △R5, and FeNO can predict lower C-ACT scales by the areas under receiver operating characteristic curves (AUCs) (0.616, 0.625, 0.609, 0.622, 0.625, and 0.714). A combination of FeNO (>20 ppb) with IOS measure significantly increased the specificity for predicting uncontrolled asthma patients compared with FeNO alone (*P* < 0.01). A multiple regression model showed that small airway parameter (R5-R20) was the strongest risk factor [OR (95% CI): 87.26 (7.67–993.31)] for uncontrolled asthma patients. Poor control with lower C-ACT scales correlated with high FeNO (*r* = −0.394), R5 (*r* = −0.106), and R5-R20 (*r* = −0.129) in asthmatic children (*P* < 0.05).

**Conclusion:**

A combined use of FeNO and IOS measurements strongly predicts childhood asthma with or without control.

## Introduction

Asthma is a chronic airway inflammatory disorder characterized by airway hypersensitivity (AHR) and reversible airflow obstruction (1, 2). Persistent airway inflammation contributes to airway remodeling and asthma progression (3–5). The Global Initiative for Asthma (GINA) has made recommendations for early diagnosis and good control based on symptoms and conventional spirometry to improve asthma outcomes (6). Impulse oscillometry (IOS) is an effort-independent method for measuring respiratory system resistance (R) and reactance (X), which is suitable for younger children unable to receive spirometry (7–9). Increasing evidence shows that IOS indicator with R5 (resistance at 5 Hz) may better predict asthma exacerbations or loss of control in children with asthma (8).

Small airway dysfunction has not been well studied in childhood asthma. Small airway obstruction can be found in asthma patients without clinical symptoms (1–3). There is evidence to support the concept that small airway dysfunction is associated with a risk factor for asthma exacerbations, asthma severity, AHR, and loss of lung function (10, 11). Thus, early assessment, recognition, and monitoring of small airway obstruction may reduce the frequency of asthma exacerbations (2, 12). The spirometry of FEF_25–75%_ is a marker of early small airway obstruction associated with lung function impairment in early adulthood (4). The IOS-defined small airway dysfunction parameters, R5–R20, X5, and AX (reactance area), are found to be correlated with poor asthma control (8, 13, 14). However, some evidence shows that spirometry measures are poorly associated with a validated asthma control questionnaire, and thus, the routine use of these small-airway dysfunction markers to detect asthma control is still debated (2, 15, 16).

Exhaled nitric oxide (NO) is produced by the bronchial epithelium *via* the activation of inducible nitric oxide synthases (17). Measurement of the fraction of exhaled NO (FeNO) has been suggested as a sensitive, non-invasive marker for monitoring Th2-mediated airway inflammation (18). Since FeNO is a surrogate marker of eosinophilic inflammation, it is logical that more severe asthmatic symptoms correlate with more airway inflammation (19). The utility of FeNO in clinical practice may assess asthma adherence and guide treatment for children with asthma (20, 21).

Currently, there is still a need to establish clinical and reference values for IOS parameters that may help identify asthma with and without control in children (1). There are limited population-based studies about using FeNO and IOS parameters for assessing asthma control with small airway dysfunction (22–25). The primary objective of this study is to investigate whether FeNO combined with IOS measures can predict childhood asthma with and without control for small airway obstruction and airway inflammation in school-age children.

## Materials and Methods

### Data Collection and Study Population

**FIGURE 1:**
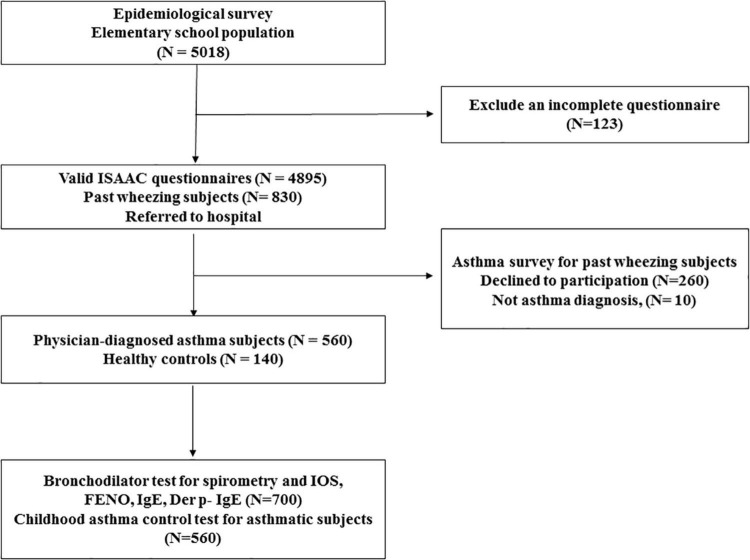
The scheme of the study flowcharts.

Study participants were recruited from the Changhua school children asthma screen and environmental factors survey and health promotion project (CARE study), a population-based cohort study in Taiwan. A total of 5,018 elementary school children aged between 6 and 12 years in Changhua County, Taiwan, were eligible for screening from July 2017 to June 2019. Students with any systemic diseases apart from asthma were excluded from participation. Child assent and parental informed consent were obtained individually from each school students and parents. The International Study of Asthma and Allergies in Childhood (ISAAC) questionnaire was translated into Chinese as a validity check for previous research (26). Video films showing scenes related to asthma symptoms, wheeze severity, and shortness of breath were administered to all school children and parents before answering the questionnaire (27). The school children took the questionnaires home, where they were filled out and signed by parents or guardians and returned to the class. The subject flow diagram is presented in Figure 1. There were 830 children with a history of wheezing by ISAAC questionnaire, and 140 age-matched randomly selected healthy children (mainly by random number table method) were consecutively referred to Changhua Christian Children’s Hospital, a tertiary care teaching hospital, for asthma diagnosis and evaluation of healthy controls. The physician-diagnosed asthma with recurrent wheeze during the previous year was established according to GINA criteria; 260 subjects refused to participate in this study, and 10 subjects were excluded because there was no evidence of asthma symptoms. Finally, 560 asthmatic children and 140 healthy controls were enrolled in this study (Figure 1). FeNO, spirometry, IOS measures, bronchial dilation test response (BDT), blood sampling for total IgE, and *Dermatophagoides pteronyssinus* (Der p)-specific IgE (Phadia, Uppsala, Sweden) were performed for each student. Each asthmatic child was assessed for asthma control using childhood asthma symptom scores (C-ACT) (19) and asthma inhalers by a pediatric respiratory specialist blinded to the above assessment results. Healthy children were defined as having no history of past wheezing, normal spirometry, and normal IgE values. The project was reviewed and approved by the Changhua County Ministry of Education and Changhua County Ministry of Health. The hospital’s institutional review board approved the study, and the parents of the children provided written informed consent (Changhua Christian Hospital IRB No. 160321 and 170320).

### Assessment of Asthma Control

The Chinese version of the C-ACT is a seven-item questionnaire for assessing asthma control with four questions answered by the child and three questions answered by the parent/caregivers (19). The score ranges from 0 to 27, and a score of 19 or less indicates inadequately controlled asthma (28).

### FeNO, Spirometry, Impulse Oscillometry, and Bronchial Dilation Test

The FeNO levels were obtained in compliance with American Thoracic Society (ATS) guidelines and measured by electrochemical analysis with a NIOX MINO device (Aerocrine AB, Solna, Sweden) (29). All children were asked to avoid food intake and physical exercise within 2 h before the test and to stop the use of inhaled corticosteroids for 1 week before the test. While a child had a cold or respiratory infection, the FeNO measurement was postponed for 1 week. FeNO was measured two times before spirometry and IOS (Astech Co., Port Washington, NY, United States). The ATS guidelines suggest that the FeNO level in children ≥20 ppb is interpreted as a higher degree of Th2-driven inflammation and increased risk of asthmatic symptoms (29, 30).

Standard spirometric maneuvers and IOS (Jaeger, Wezburg, Germany) were performed in accordance with ATS and European Respiratory Society standards (31, 32). All subjects withheld the use of short- and long-acting bronchodilators for 12 h before the study. Children performed IOS and spirometry before and after 200 μg salbutamol inhalation *via* a spacer according to a standardized protocol (33–37). IOS was performed before spirometry in each child due to the possible effects of forced expiratory maneuvers on bronchial motor tone. Spirometric reference values (38) in this study were using up-to-date reference equations in healthy Taiwanese children because Global Lung Function Initiative (GLI)-2012 reference equations were not closely fitted in a contemporary Taiwanese child population. Percent predicted normal values of spirometry (best of 3 repeated maneuvers) were used for analyses. FEV1, FVC, and FEF_25–75_ were reported as% predicted, and FEV1/FVC ratio was reported as raw values only. Among IOS parameters, R5 and Zrs (pulmonary impedance at 5 Hz) reflect total airway resistance. The differences between R5 and R20 (R5–R20), X5, and AX reflect changes in the degree of obstruction of peripheral airways. Currently, the recommended thresholds for defining a positive bronchodilator response (BDT) for children are an increase of 12% in FEV1 and a decrease of 40% in R5 (31, 32).

### Statistical Analysis

As most continuous variables revealed a positively skewed distribution, continuous variables are presented as the median and interquartile range (IQR), while categorical variables are presented as absolute (n) and relative (%) frequencies. The case-control study enrolled asthma patients and control subjects at a ratio of 4:1. The minimum sample size was calculated with 95% confidence level, maximum marginal error (5%), and 80% power for testing the prevalence of asthma based on the sensitivity (85%) and comparing two diagnostic tasks. In this study, the AUC of R5 value to predict asthma diagnosis was 0.668. The minimum sample size required 107 subjects for the asthma group and 27 subjects for the control group using MedCalc Statistical Software, version 19.7.2 (MedCalc Software Ltd., Ostend, Belgium). Finally, this study enrolled 560 asthmatic children and 140 healthy controls, and the statistical test power reached 0.99. To assess whether clinical background variables differed between asthmatic patients and healthy subjects, the chi-square test or Fisher’s exact test was used for categorical data, and the Mann–Whitney *U*-test was used for continuous data. Different asthma control (C-ACT) groups were compared using the Kruskal–Wallis test. The relationship between variables was evaluated using Spearman’s rank correlation coefficient. Receiver operating characteristics (ROC) curve analysis was performed to evaluate the diagnostic ability in order to differentiate asthma and healthy controls among children. The discriminative power of the single or combined measurements was determined by ROC curves: the area under the curve (AUC), sensitivity, specificity, positive predictive value (PPV), negative predictive value (NPV), positive likelihood ratio (LR +), and negative likelihood ratio (LR-). Univariate and multiple logistic regression analyses were also used to compare different diagnostic tools (FeNO, IOS, and spirometry variables) to determine which one was more predictive. All data were analyzed using IBM SPSS Statistics for Windows, Version 22.0 (IBM Corp., Armonk, NY, United States). A *P*-value less than 0.05 was considered to be statistically significant.

## Results

### General Characteristics of Subjects

Demographic data, spirometry, IOS, and C-ACT values are summarized in Table 1. Characteristics of the cohort with 700 subjects revealed that the median age of the patients was 9.0 years (IQR range, 8–11 years). No statistical differences between the asthmatic children and healthy controls were detected in age, sex, height, and weight (*P* > 0.05). Their average C-ACT scores were 26 (IQR range, 24–27) in asthmatic children, and 39.1% of the asthmatic patients had an acute exacerbation in the past year, including 5.4% of patients who had a previous emergency department visit and 0.9% of patients with a history of hospitalization in the past year (Table 1). For asthma control treatment, 40 (7.1%) patients received ICS, 5 (0.9%) patients received a long-acting beta2-agonist in a combined formulation with ICS (ICS/LABA), and 70 (12.5%) patients received leukotriene modifiers (Table 1).

**TABLE 1:** Subject characteristics.

	Asthma (*n* = 560)		Healthy controls (*n* = 140)		*P*-value
	Median	IQR	Median	IQR	
Age (year)	9.00	8.00 to 11.00	9.00	8.00 to 11.00	0.898
Male (n,%)	314.00	(56.1%)	69.00	(49.3%)	0.149
Height (cm)	138.00	127.00 to 147.00	138.00	129.00 to 149.80	0.345
Weight (kg)	33.50	26.00 to 44.00	35.00	27.30 to 43.50	0.570
BMI	17.63	15.79 to 20.89	17.67	15.75 to 20.33	0.902
C-ACT scores	26.00	24.00 to 27.00			
Acute exacerbations in the past year (n,%)	219.00	(39.1%)			
Previous ED visit (n,%)	30.00	(5.4%)			
Previous hospitalized in the past year (n,%)	5.00	(0.9%)			
Difficult to control/severe asthma (n,%)	5.00	(0.9%)			
**Home asthma controllers (n,%)**					
ICS	40.00	(7.1%)			
Combined ICS/LABA	5.00	(0.9%)			
Leukotriene modifiers	70.00	(12.5%)			
FENO (ppb)	38.00	22.25 to 56.15	13.00	6.00 to 17.00	<0.001
Total IgE (IU)	449.00	242.00 to 930.00	40.00	13.60 to 58.00	<0.001
Der p (IU/ml)	66.00	23.00 to 100.00	0.00	0.00 to 1.00	<0.001
**Spirometry parameters, baseline**					
FVC (% predicted)	93.92	85.53 to 102.24	94.54	87.05 to 105.69	0.146
FEV1 (% predicted)	94.97	86.45 to 102.70	98.23	90.73 to 107.34	0.001
FEV1/FVC (%)	101.19	95.80 to 106.39	103.41	97.86 to 108.97	0.004
FEF_25–75_ (% predicted)	80.49	66.67 to 97.05	88.25	71.72 to 102.60	0.001
PEFR (% predicted)	91.93	80.66 to 102.59	95.27	85.92 to 105.69	0.010
**Bronchodilator response (spirometry)**					
△FEV1 (%)	1.51	−1.42 to 4.63	1.13	−1.72 to 3.56	0.179
△FEV_25–75_ (%)	10.50	2.36 to 21.62	9.84	2.93 to 20.71	0.665
**IOS metrics parameters, baseline**					
Zrs (kPa L^–1^ s)	0.75	0.62 to 0.91	0.64	0.54 to 0.77	<0.001
R5 (kPa L^–1^ s)	0.72	0.60 to 0.87	0.62	0.52 to 0.73	< 0.001
R5-R20 (kPa L^–1^ s)	0.17	0.10 to 0.26	0.10	0.05 to 0.17	< 0.001
X5 (kPa L^–1^ s)	−0.18	−0.25 to −0.14	−0.16	−0.20 to −0.13	< 0.001
Ax (kPa/L)	1.36	0.75 to 2.32	0.78	0.45 to 1.36	< 0.001
Fres. (^–1^ s)	19.37	16.00 to 22.68	16.22	12.46 to 19.75	< 0.001
**Bronchodilator response (IOS)**					
△R5 (%)	15.02	7.31 to 22.22	9.51	1.68 to 17.73	< 0.001
△R5-R20 (%)	30.50	8.08 to 50.00	21.37	0.00 to 44.54	0.008
△AX (%)	36.00	15.69 to 52.46	23.84	3.36 to 42.87	< 0.001

*ED, emergency department; ICS, inhaled corticosteroid; LABA, long-acting 2-adrenergic agonists; FENO, fraction exhaled nitric oxide; Der p, Dermatophagoides pteronyssinus; IOS, impulse oscillometry; Fres, resonant frequency; R5, resistance at 5 Hz; R20, respiratory resistance at 20 Hz; X5, respiratory reactance at 5 Hz; AX, area of reactance; Δ, percentage of bronchodilator response; IQR, interquartile range represents the distance between the 25th percentile and 75th percentile. P-value by Mann–Whitney U test.*

All participants completed FeNO, spirometric tests, IOS, and BDT. Of the enrolled 560 asthmatic children, 77 asthmatic children (13.8%) had an obstructive airway pattern (FEV1 < 80%), while 102 asthmatic patients (18.2%) had a small airway pattern (FEF25–75 < 60%) and 26 asthmatic patients (4.6%) had a BDT response by spirometry criteria for asthma. FeNO, total IgE, Der p-specific IgE, spirometry parameters (FEV1, FEV1/FVC, FEF_25–75_, PEFR), and IOS parameters (Zrs, R5, R5-R20, X5, Ax, Fres) all showed a significant difference in the asthmatic subjects compared with the controls (*P* < 0.01, Table 1). After inhaling salbutamol, no significant post-bronchodilator changes in FEV1 and FEF_25–75_ were found in all asthmatic children compared with the controls (*P* > 0.05, Table 1). However, asthmatic patients receiving ICS had a significant post-bronchodilator response in FEV1 values when compared with asthmatic patients not receiving ICS (*P* < 0.01) (Supplementary Table 1). Asthmatic patients had a significant post-bronchodilator response in IOS values with R5, R5-R20, and Ax change when compared with healthy controls (*P* < 0.01) (Table 1). In addition, asthmatic subjects receiving ICS had a significant difference in the IOS parameters (Zrs, R5, X5, Ax, Fres) and post-bronchodilator changes in R5, R5-R20, and Ax compared with asthmatic patients not receiving ICS (*P* < 0.05) (Supplementary Table 1).

### Impulse Oscillometry Measures Improved the Diagnostic Accuracy of FeNO for Asthma

Using spirometry as the gold standard for asthma detection, the discriminating performance of IOS was investigated. Because there are no standard reference values of IOS for normal children in this community, the raw values of IOS were used instead of the percentage of predicted values. Normal IOS cutoff values defined R5, R5-R20, Ax, Zrs, Fres > 95th percentile or X5 values < 5th percentile based on healthy controls, and FEV1 > 80%, MMEF > 60%, and BDT < 12% by asthma criteria, which are shown in Supplementary Table 2. The cutoff values for asthma diagnosis with R5 ≥ 0.89 kPa L/s (AUC/sensitivity/specificity: 0.668/23.4%/95%) and FeNO levels (≥ 20 ppb) (AUC/sensitivity/specificity: 0.886/80.7%/85.0%) are shown in Supplementary Table 3. A multiple regression model showed that major IOS predictors for asthma diagnosis were R5-R20, R5, and Zrs (all *P* < 0.01) (Table 2).

**TABLE 2:** Logistic regression analysis of asthma diagnosis.

	Univariate analysis (crude)			Multiple analysis (adjusted)*		
(*n* = 700)	Odds ratio	95% CI	*P*-value	Odds ratio	95% CI	*P*-value
FENO (ppb)	1.139	1.109-1.169	< 0.001	1.171	1.134-1.209	< 0.001
R5 (kPa L^–1^ s)	35.494	10.899-115.597	< 0.001	683.987	80.063-5843.370	< 0.001
R5-R20 (kPa L^–1^ s)	1345.561	156.339-11580.845	< 0.001	6079.579	314.912-117370.009	< 0.001
X5 (kPa L^–1^ s)	0.003	0.000-0.048	< 0.001	0.001	0.000-0.033	< 0.001
Ax (kPa/L)	2.040	1.593-2.612	< 0.001	2.560	1.805-3.629	< 0.001
Fres. (^–1^ s)	1.131	1.085-1.179	< 0.001	1.172	1.105-1.243	< 0.001
Zrs (kPa L^–1^ s)	25.888	8.433-79.472	< 0.001	441.418	56.605-3442.255	< 0.001
△R5 (%)	1.027	1.012-1.041	< 0.001	1.049	1.028-1.070	< 0.001
△R5-R20 (%)	1.001	0.999-1.003	0.438	1.002	1.000-1.005	0.066
△AX (%)	1.007	1.002-1.012	0.004	1.013	1.007-1.020	< 0.001
FEV1 (% predicted)	0.975	0.961-0.990	0.001	0.975	0.956-0.994	0.012
FVC (% predicted)	0.985	0.970-1.000	0.048	0.981	0.961-1.003	0.084
FEV1/FVC (%)	0.966	0.943-0.989	0.004	0.970	0.941-0.999	0.046
FEF25–75 (% predicted)	0.987	0.979-0.995	0.001	0.990	0.980-1.001	0.065
PEFR (% predicted)	0.986	0.975-0.997	0.014	0.988	0.973-1.003	0.106
△FEV1 (%)	1.017	0.987-1.047	0.268	1.003	0.967-1.040	0.881
△FEV25–75 (%)	1.003	0.993-1.012	0.591	0.998	0.985-1.011	0.743
Total IgE (IU)	1.053	1.040-1.066	< 0.001	1.046	1.033-1.060	< 0.001
Der p (IU/ml)	1.280	1.183-1.385	< 0.001	1.240	1.137-1.353	< 0.001

*Adjusted for age, gender, BMI, and FENO.

Overall, FeNO above 20 ppb combined with IOS and spirometry measurements had a higher specificity for asthma diagnosis than FeNO alone (all *P* < 0.01, Table 3).

**TABLE 3:** Predictive values of FENO (> 20 ppb) combined with IOS and spirometry measurements for asthma diagnosis.

		Cut-off	Criterion values and coordinates of ROC curve						Area under the ROC curve			
Variable		Value	Sensitivity	Specificity	PPV	NPV	LR +	LR-	AUC	SE	95% CI	*P*-value
FENO > 20 combined with												
Zrs (kPa L^–1^ s)	≥	0.93	0.141	1.000	1.000	0.225	–	0.859	0.896	0.016	0.864-0.927	< 0.001
R5 (kPa L^–1^ s)	≥	0.89	0.143	1.000	1.000	0.226	–	0.857	0.898	0.016	0.867-0.929	< 0.001
R5-R20 (kPa L^–1^ s)	≥	0.29	0.121	0.993	0.986	0.220	17.000	0.885	0.891	0.018	0.856-0.926	< 0.001
X5 (kPa L^–1^ s)	≤	−0.26	0.139	0.986	0.975	0.223	9.750	0.873	0.868	0.019	0.831-0.906	< 0.001
Ax (kPa/L)	≥	2.30	0.170	0.993	0.990	0.230	–	0.836	0.887	0.019	0.850-0.923	< 0.001
Fres. (^–1^ s)	≥	23.88	0.107	1.000	1.000	0.219	–	0.893	0.882	0.018	0.848-0.917	< 0.001
△R5 (%)	≥	40.0	0.046	1.000	1.000	0.208	–	0.954	0.878	0.017	0.845-0.911	< 0.001
△R5-R20 (%)	≥	165.0	0.011	1.000	1.000	0.202	–	0.989	0.858	0.020	0.819-0.896	< 0.001
△AX (%)	≥	99.0	0.021	1.000	1.000	0.203	–	0.979	0.869	0.019	0.831-0.907	< 0.001
FEV1 (% predicted)	≤	80.00	0.105	1.000	1.000	0.218	–	0.895	0.839	0.019	0.801-0.877	< 0.001
FVC (% predicted)	≤	80.00	0.105	1.000	1.000	0.218	–	0.895	0.822	0.021	0.781-0.864	< 0.001
FEV1/FVC (%)	≤	80.00	0.018	1.000	1.000	0.203	–	0.982	0.851	0.018	0.815-0.886	< 0.001
FEF_25–75_ (% predicted)	≤	60.00	0.132	1.000	1.000	0.224	–	0.868	0.851	0.018	0.817-0.886	< 0.001
PEFR (% predicted)	≤	80.00	0.177	0.979	0.971	0.229	8.250	0.841	0.840	0.020	0.801-0.879	< 0.001
△FEV1 (%)	≥	12.00	0.032	0.993	0.947	0.204	4.500	0.975	0.832	0.020	0.793-0.871	< 0.001
△FEV_25–75_ (%)	≥	30.00	0.116	0.979	0.956	0.217	5.417	0.903	0.832	0.019	0.793-0.870	< 0.001

*PPV, positive predictive value; NPV, negative predictive value; LR +, positive likelihood ratio; LR-, negative likelihood ratio; AUC, area under the curve.*

### Characteristics and Predictive Values of FeNO and Impulse Oscillometry Measurements in Uncontrolled Asthmatic Patients

In this study, 226 asthmatic patients (40.4%) were well controlled, 289 asthmatic patients (51.6%) were partially controlled (ACT: 20–26), and 45 asthmatic patients (8%) were non-controlled, based on ACT scores < 20 (Table 4). Table 5 compares the FeNO, absolute IOS values, percent predicted spirometric values, and BDT values among uncontrolled, partially controlled, and totally controlled asthmatic children. IOS and spirometric measures were significantly abnormal in subjects whose symptoms remained uncontrolled to a greater extent than the total controlled asthmatic subjects (all *P* < 0.05) (Table 4). FeNO, spirometric values, and IOS parameters with R5, R5-R20, AX, Zrs, and △R5 were useful to distinguish uncontrolled asthmatic children between partially or totally controlled asthmatic children (*P* < 0.05) (Table 4).

**TABLE 4:** FENO, IOS, and spirometry measurements between the total controlled, partial controlled, and uncontrolled groups among asthmatic children.

	Uncontrolled (< 20) (*n* = 45)		Partial controlled (20–26) (*n* = 289)		Total controlled (27) (*n* = 226)	
	Median	IQR	Median	IQR	Median	IQR
C-ACT score	16.0*^#^	13.0-17.0	25.0*	23.0-26.0	27.0	27.0-27.0
FENO (ppb)	54.0*^#^	36.0-104.0	43.3*	28.7-62.0	26.0	13.3-43.5
Total IgE (IU)	493.0	312.0-944.0	503.0	255.0-1016.5	387.0	216.0-713.0
Der p (IU/ml)	83.0*	26.0-100.0	75.0*	30.8-100.0	47.0	17.1-100.0
FVC (% predicted)	87.6*^#^	77.2-99.5	92.5*	84.1-101.3	96.4	89.6-104.2
FEV1 (% predicted)	80.5*^#^	68.3-95.2	92.9*	84.9-100.7	98.1	91.2-106.1
FEV1/FVC (%)	95.9*^#^	89.9-102.8	100.7*	95.1-106.3	102.6	98.0-106.5
FEF_25–75_ (% predicted)	56.6*^#^	42.3-76.2	78.6*	63.2-95.2	85.5	73.3-102.4
PEFR (% predicted)	81.2*^#^	70.7-92.4	90.5*	80.8-100.4	94.3	83.5-105.4
△FEV1 (%)	7.6*^#^	3.4-13.2	1.9*	−1.3-4.9	0.6	−2.1-2.9
△FEV_25–75_ (%)	25.1*^#^	11.7-43.5	10.7*	2.9-22.7	8.2	1.2-18.0
R5 (kPa L^–1^ s)	0.8*^#^	0.6-0.9	0.7	0.6-0.9	0.7	0.6-0.9
R5-R20 (kPa L^–1^ s)	0.2*^#^	0.1-0.3	0.2	0.1-0.3	0.2	0.1-0.2
X5 (kPa L^–1^ s)	−0.2*	−0.3-−0.2	−0.2	−0.2-−0.1	−0.2	−0.2-−0.1
Ax (kPa/L)	2.2*^#^	1.1-3.0	1.4	0.8-2.2	1.2	0.7-2.2
Fres. (^–1^ s)	20.9*	18.0-24.9	19.5	16.6-22.6	18.6	15.2-22.4
Zrs (kPa L^–1^ s)	0.9*^#^	0.7-1.0	0.8	0.6-0.9	0.7	0.6-0.9
△R5 (%)	21.2*^#^	13.4-27.4	14.0	7.3-21.3	15.1	6.6-22.2
△R5-R20 (%)	39.5	5.3-50.0	27.8	7.8-48.4	33.7	10.0-54.2
△AX (%)	41.3	15.8-53.7	35.8	10.8-52.4	35.6	17.5-52.2

IQR, interquartile range represents the distance between the 25th percentile and 75th percentile. P-value by Kruskal–Wallis test (*P < 0.05 uncontrolled and partial controlled group vs. total controlled group, ^#^P < 0.05 uncontrolled group vs. partially controlled group).

**TABLE 5:** Predictive values for uncontrolled asthma among asthmatic children between FENO, IOS, and spirometry measurements.

		Cut-off	Criterion values and coordinates of ROC curve					ROC curve for FENO > 20 ppb combined with		
			
Variable		Value	Sensitivity	Specificity	AUC	*P*-value	Sensitivity	Specificity	AUC	*P*-value
FENO (ppb)	≥	20.00	0.978	0.209	0.714	< 0.001				
Zrs (kPa L^–1^ s)	≥	0.93	0.333	0.781	0.617	0.010	0.356	0.878	0.722	< 0.001
R5 (kPa L^–1^ s)	≥	0.89	0.378	0.777	0.616	0.010	0.356	0.876	0.721	< 0.001
R5-R20 (kPa L^–1^ s)	≥	0.29	0.244	0.817	0.625	0.005	0.244	0.889	0.718	< 0.001
X5 (kPa L^–1^ s)	≤	−0.26	0.378	0.809	0.609	0.015	0.356	0.880	0.709	< 0.001
Ax (kPa/L)	≥	2.30	0.257	0.950	0.622	0.007	0.444	0.854	0.716	< 0.001
Fres. (^–1^ s)	≥	23.88	0.333	0.847	0.606	0.018	0.311	0.911	0.702	< 0.001
△R5 (%)	≥	40.00	0.044	0.974	0.625	0.006	0.044	0.988	0.720	< 0.001
△R5-R20 (%)	≥	165.00	0.022	0.994	0.528	0.530	0.022	0.998	0.647	0.001
△AX (%)	≥	99.00	0.000	0.998	0.528	0.530	0.000	1.000	0.650	0.001
FEV1 (% predicted)	≤	80.00	0.489	0.899	0.734	< 0.001	0.467	0.926	0.779	< 0.001
FVC (% predicted)	≤	80.00	0.333	0.893	0.640	0.002	0.333	0.915	0.699	< 0.001
FEV1/FVC (%)	≤	80.00	0.111	0.986	0.667	< 0.001	0.111	0.990	0.731	< 0.001
FEF_25–75_ (% predicted)	≤	60.00	0.556	0.864	0.754	< 0.001	0.533	0.903	0.800	< 0.001
PEFR (% predicted)	≤	80.00	0.467	0.780	0.686	< 0.001	0.444	0.847	0.750	< 0.001
△FEV1 (%)	≥	12.00	0.267	0.982	0.785	< 0.001	0.267	0.988	0.818	< 0.001
△FEV_25–75_ (%)	≥	30.00	0.422	0.871	0.732	< 0.001	0.422	0.911	0.788	< 0.001

*AUC, area under the curve.*

All discriminative properties of the FeNO, oscillometric, and spirometric variables in predicting the uncontrolled asthma are shown using AUC analysis (Table 5). IOS parameters with R5, R5-R20, X5, Ax, △R5, and FeNO could predict uncontrolled asthma with estimated AUCs (0.616, 0.625, 0.609, 0.622, 0.625, and 0.714) (all *P* < 0.05) (Table 5 and Supplementary Table 4). FeNO (>20 ppb) combined with IOS measures (R5, R5-R20, X5, Ax, and △R5) improved the AUCs values (0.721, 0.718, 0.709, 0.716, and 0.720) in predicting the uncontrolled asthma with sensitivity (35.6%, 24.4%, 35.6%, 44.4%, and 4.4%) and specificity (87.6%, 88.9%, 88%, 85.4%, and 98.8%) (all *P* < 0.01) (Table 5 and Supplementary Table 5).

### Associations Between FeNO, Spirometric and Impulse Oscillometry Measurements, and Childhood Asthma Control Test Scores

**FIGURE 2:**
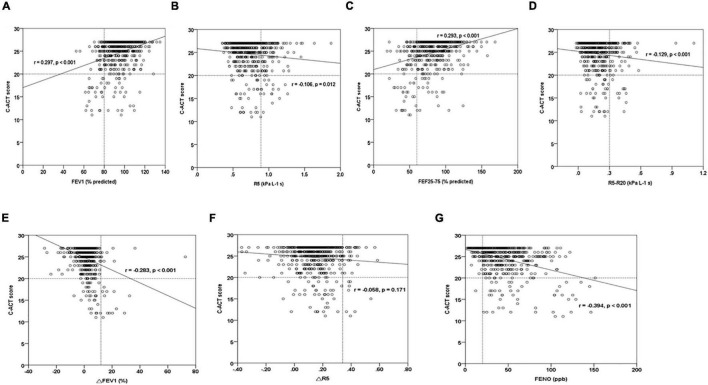
The graph of the C-ACT score relationships among FeNO, spirometry, and IOS parameters in asthmatic children. **(A,B)** C-ACT scores vs. obstructive airway disease parameters (FEV1, R5), **(C,D)** C-ACT scores vs. small airway disease parameters (FEF_25–75_, R5-R20), **(E,F)** C-ACT scores vs. bronchodilator response parameters (△R5, △FEV1), **(G)** C-ACT scores vs. FeNO. *r*, Spearman’s rho correlation coefficient.

A multiple regression model showed that the presence of IOS defined-small airway obstructive marker, R5-R20, represented the strongest risk factor [odds ratio (95% CI): 87.26 (7.67–993.31)] for uncontrolled asthmatic children (*P* < 0.01) (Table 6). Poor asthma control with lower C-ACT values had a significant correlation among obstructive airway disease pattern (FEV1%, *r* = 0.297; R5, *r* = −0.106), small airway obstruction (FEF_25–75%_, *r* = 0.293; R5-R20, *r* = −0.129), post-bronchodilator FEV1% change (*r* = −0.283), and FeNO levels (*r* = −0.394) in asthmatic children (all *P* < 0.01) (Figure 2). Further analysis showed that FeNO values were correlated negatively with FEV1 (%) (*r* = −0.09; *P* = 0.02) and R5 (*r* = −0.102; *P* < 0.01), suggesting that obstructive airway disease pattern in asthmatic children may be associated with eosinophilic airway inflammation (Supplementary Figure 1).

**TABLE 6:** Logistic regression analysis of uncontrolled asthma among asthmatic children.

	Univariate analysis (crude)			Multiple analysis (adjusted)*		
(*n* = 560)	Odds ratio	95% CI	*P*-value	Odds ratio	95% CI	*P*-value
FENO (ppb)	1.029	1.019-1.039	< 0.001	1.030	1.020-1.041	< 0.001
R5 (kPa L^–1^ s)	4.806	1.316-17.545	0.017	37.747	6.809-209.274	< 0.001
R5-R20 (kPa L^–1^ s)	11.517	1.500-88.402	0.019	87.256	7.673-992.309	< 0.001
X5 (kPa L^–1^ s)	0.044	0.002-0.877	0.041	0.003	0.000-0.123	0.002
Ax (kPa/L)	1.263	1.049-1.520	0.014	1.490	1.198-1.853	< 0.001
Fres. (^–1^ s)	1.076	1.011-1.145	0.021	1.110	1.035-1.189	0.003
Zrs (kPa L^–1^ s)	4.405	1.292-15.019	0.018	30.214	6.012-151.849	< 0.001
△R5 (%)	1.036	1.012-1.061	0.003	1.048	1.022-1.075	< 0.001
△R5-R20 (%)	1.003	0.996-1.009	0.441	1.005	0.997-1.012	0.197
△AX (%)	1.002	0.993-1.011	0.688	1.005	0.995-1.015	0.310
FEV1 (% predicted)	0.922	0.898-0.947	< 0.001	0.911	0.884-0.939	< 0.001
FVC (% predicted)	0.954	0.930-0.978	< 0.001	0.947	0.922-0.973	< 0.001
FEV1/FVC (%)	0.926	0.895-0.958	< 0.001	0.914	0.879-0.949	< 0.001
FEF_25–75_ (% predicted)	0.955	0.940-0.970	< 0.001	0.949	0.932-0.966	< 0.001
PEFR (% predicted)	0.959	0.939-0.979	< 0.001	0.947	0.925-0.970	< 0.001
△FEV1 (%)	1.134	1.081-1.191	< 0.001	1.132	1.078-1.189	< 0.001
△FEV_25–75_ (%)	1.029	1.015-1.043	< 0.001	1.032	1.017-1.048	< 0.001
Total IgE (IU)	1.000	1.000-1.000	0.250	1.000	1.000-1.000	0.961
Der p (IU/ml)	1.005	0.996-1.013	0.285	0.994	0.984-1.004	0.231

*Adjusted for age, gender, BMI, and FENO.

## Discussion

This study provides further evidence that IOS and spirometry are comparable in assessing control in children with asthma. The findings show clinically significant dysfunction of small airway obstruction and its reversibility, as well as FeNO in the asthmatic children with poor control. We found that small airway dysfunction, bronchodilator effects, and FeNO values were higher in the uncontrolled asthmatic patients than in partial and total controlled asthmatic subjects. FeNO levels had a significantly negative correlation with FEV1 and R5, adding more evidence that airway obstruction is associated with eosinophilic airway inflammation. FENO > 20 ppb, as recommended by the ATS guidelines, has a high sensitivity (97.8%) but a weak specificity (20.9%) for detecting asthmatic patients with lower C-ACT scales. The combined use of FeNO and IOS measures was confirmed to improve the predictive values with specificity above 85% for uncontrolled asthmatic children. One strength of our prospective cohort study is the large group of pediatric patients with asthma. Mild asthma accounts for the majority of asthma cases in this community-based population survey. Impulse oscillometry is not widely used at present in part due to the lack of standard reference values for healthy and asthmatic children. We have shown that IOS cutoff values were defined by spirometry criteria with FEV1 > 80%, MMEF > 60%, and BDR < 12% based on asthmatic children with current wheeze and health children as shown in Supplementary Table 2. Spirometry could not be easily performed in younger children with asthma. We suggest that physicians may choose IOS instead of spirometry, especially for the busy clinic setting.

Previous studies have reported that small airway dysfunction is associated with future loss of asthma control even when treated with ICS (11). A prospective birth cohort study showed that IOS-defined small airway indicators with R5-R20 at 16 years of age are related to persistent asthma in adolescence (39). There is also evidence that IOS parameters (AX, R5–R20) are clinically useful in identifying mild-to-moderate controlled asthma children at risk to lose control after 8–12 weeks follow-up visit (40). In this study, R5-R20 is the strongest risk factor for uncontrolled asthmatic children in multiple regression analysis. This is consistent with previous findings that IOS-defined peripheral airway impairment phenotype is useful in identifying uncontrolled asthmatic patients.

Accumulated evidence suggested that lack of asthma control can be explained by persistent inflammation and narrowing in the lung’s peripheral airways (1–3). A longitudinal birth cohort study showed that IOS-defined small airway dysfunction and FeNO seem to be a feature related to eosinophilic airway inflammation in adolescents with asthma (41). A previous study reported that FEF_25–75%_ combined with FeNO can improve the predictive value for adults with cough-variant asthma (42). This study showed that a combined FeNO and small airway function measurement provided a better prediction of lower C-ACT scores with high AUCs. These results suggest that eosinophilic airway inflammation is a more important determinant of small airway dysfunction in asthmatic control.

Persistent bronchodilator response following diagnosis of asthma even in patients with regular asthma treatment is considered a determinant of increased future risk of poor asthma control (43). Recent evidence confirms that post-bronchodilator IOS response is superior to spirometry in detecting future loss of asthma control (43, 44). We also found a higher percentage of positive BDT in uncontrolled asthmatic children. In addition, FeNO combined with either R5 or FEV1 BDT parameters had the same high specificity (98.8%) prediction of asthmatics with poor control.

In this study, we did not conduct the bronchoprovocation tests for children because methacholine challenge tests are time-consuming and inconvenient and may carry a risk of bronchial spasm. For patients who have asthma symptoms but normal lung functions, determination of AHR with methacholine may help to ascertain the diagnosis of asthma (45). The GINA guidelines do not specify a bronchodilator range for BDR testing and simply recommend a salbutamol dose of 200–400 μg (6). Several studies (33, 34) on asthmatic children used 2 puffs (200 μg) of salbutamol for oscillometric BDR testing, because oscillometric lung function is more sensitive than conventional spirometry in children (35). The lower dose of 200 μg salbutamol in our protocol reduced the bronchodilator response for spirometry; however, asthmatic children still had a significant post-bronchodilator response in IOS values with R5, R5-R20, and Ax change when compared with healthy controls (*P* < 0.01) (Table 1). Although 400 μg inhaled salbutamol exhibited maximal bronchodilating effects in children, the bronchodilator dose with 200 μg of salbutamol exhibits a minimal side effect with palpitation and tremor during routine spirometric evaluations. This limitation of the study may lead to underestimating the significance of the relationship between airway reversibility and asthma control.

Asthma in childhood was a heterogeneous disease with variable clinical manifestations. Of the enrolled, 560 asthmatic children are mainly diagnosed and treated by primary care physicians; 20.5% asthmatic children were actually on regular controller treatment; 77 subjects had airway obstruction; 102 subjects had small airway obstruction; 26 subjects had BDT response; and 45 subjects had uncontrolled C-ACT scores in this population-based epidemiological survey. Mild asthmatic children with total controlled scores (*n* = 266) may attribute to a weak correlation among lower C-ACT values, obstructive airway disease pattern, small airway obstruction, BDR response, and FeNO levels (Figure 2). It is possible that not all the symptomatic children present with chronic asthma, especially those with isolated cough. Further study is needed in a population of children presenting with respiratory symptoms that might be related to asthma.

In conclusion, our study highlights that R5-R20 is a particularly important IOS-defined small airway parameter associated with the risk of diagnosis and controlled status of asthma. The clinical significance of small conducting airway dysfunction in uncontrolled asthma and its association with increased levels of FeNO might explain the pathophysiology of pediatric asthma. The data support the importance of recognizing small airway dysfunction because it enables the physician to consider treating the small airway region. C-ACT is a useful tool for assessing asthma control, although it should be better evaluated together with lung function, especially including IOS and FeNO. The combination with FeNO and IOS measurement may be a practical application for identifying uncontrolled asthma and the need for further management of the disease in children who are unable to accept spirometry.

## Data Availability Statement

The raw data supporting the conclusions of this article will be made available by the authors, without undue reservation.

## Ethics Statement

The studies involving human participants were reviewed and approved by Changhua Christian Hospital IRB Nos. 160321 and 170320. Written informed consent to participate in this study was provided by the participants’ legal guardian/next of kin.

## Author Contributions

L-ML, KY, C-HL, J-KK, J-WC, M-SL, C-YL, T-IC, and Y-GT conceptualized the study and collected the data. L-ML, Y-JC, and Y-GT analyzed the data and drafted the manuscript. KY and Y-GT designed the study and revised the manuscript critically for important intellectual content. All authors read and approved the final manuscript.

## Conflict of Interest

The authors declare that the research was conducted in the absence of any commercial or financial relationships that could be construed as a potential conflict of interest.

## Publisher’s Note

All claims expressed in this article are solely those of the authors and do not necessarily represent those of their affiliated organizations, or those of the publisher, the editors and the reviewers. Any product that may be evaluated in this article, or claim that may be made by its manufacturer, is not guaranteed or endorsed by the publisher.

## Abbreviations

ATS: American Thoracic Society
AHR: airway hyperresponsiveness
BDT: Bronchial dilation test
C-ACT: childhood asthma control test
Der p: *Dermatophagoides pteronyssinus*
FeNO: fraction exhaled nitric oxide
FEV1: forced expiratory volume in 1 s
FVC: forced vital capacity
FEF_25–75%_: forced expiratory flow between 25 and 75%
GINA: global initiative for asthma
ICS: inhaled corticosteroid
IOS: impulse oscillometry
Fres: resonant frequency.
