# Supplementary material for: Small Airway Dysfunction Measured by Impulse Oscillometry and Fractional Exhaled Nitric Oxide Is Associated With Asthma Control in Children
Source: Front Pediatr. 2022 Jun 17;10:877681. doi: 10.3389/fped.2022.877681 (PMC9247317; doi:10.3389/fped.2022.877681)
Supplement: Supplementary file 1 [file Image_1.pdf]

**Supplementary Figure 1. The graph of the relationships among FeNO, spirometry and IOS parameters in children. FeNO (ppb) versus (a) FEV1 (% predicted), (b) $FEF_{25-75}$ (% predicted), (c) △FEV1(%), (d) R5 (kPa $L^{-1}$ s), (e) R5-R20 (kPa $L^{-1}$ s), and (f) △R5 (%). r, Spearman's rho correlation coefficient**

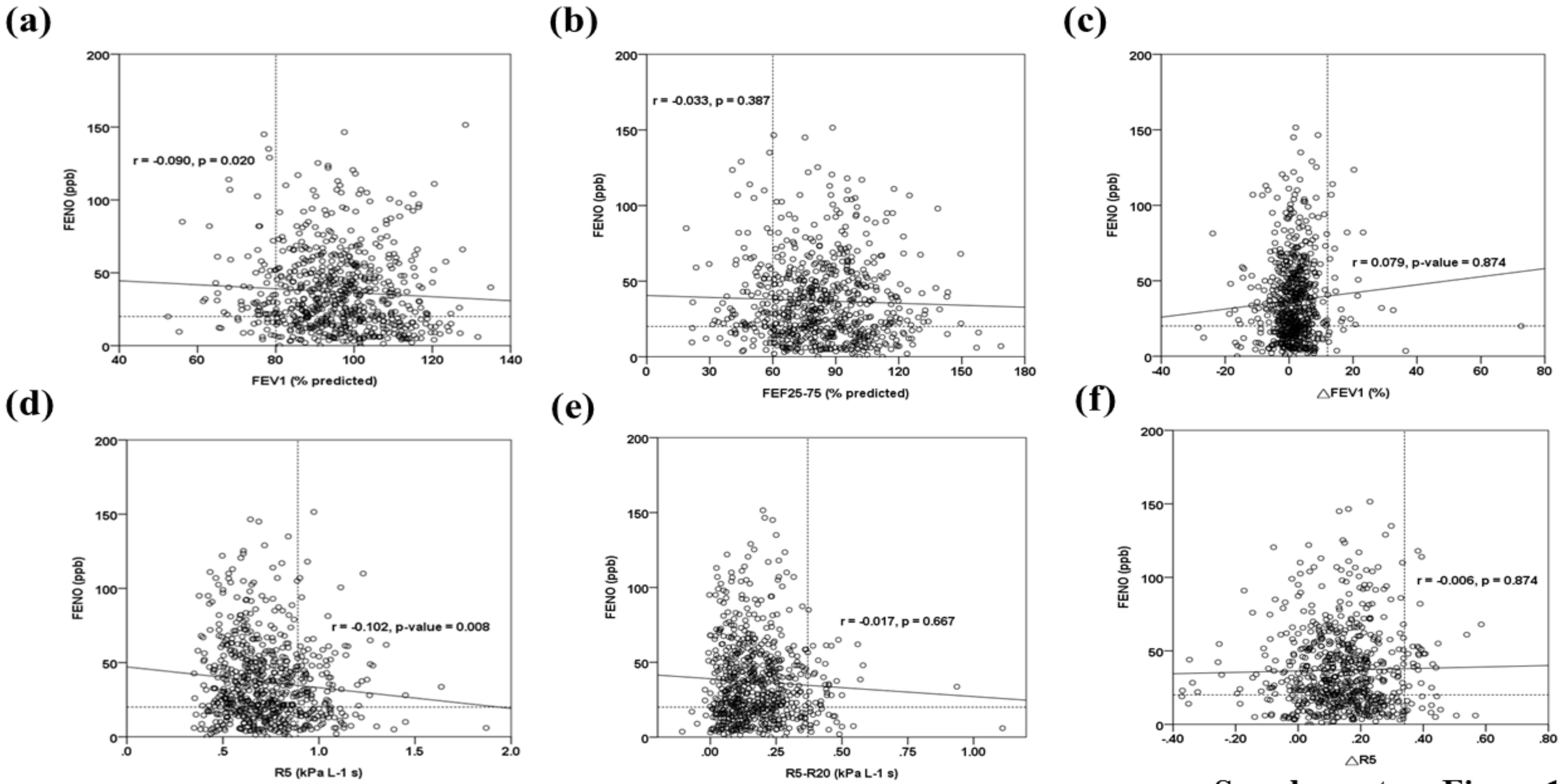


**Supplementary Figure 1**
