# Supplementary material for: Small Airway Dysfunction Measured by Impulse Oscillometry and Fractional Exhaled Nitric Oxide Is Associated With Asthma Control in Children
Source: Front Pediatr. 2022 Jun 17;10:877681. doi: 10.3389/fped.2022.877681 (PMC9247317; doi:10.3389/fped.2022.877681)
Supplement: Supplementary file 2 [file Table_1.pdf]

Supplementary 1. Lung function and FENO differences between the asthmatic patients with/without ICS.

	Asthma with ICS (n=45)				Asthma without ICS (n=515)				P-value
	Median	IQR			Median	IQR			
Spirometry parameters, baseline									
FVC (% predicted)	92.90	83.34	to	101.89	93.90	85.96	to	102.43	0.291
FEV1 (% predicted)	91.80	80.61	to	101.64	95.30	86.80	to	102.73	0.054
FEV1/FVC (%)	98.20	93.15	to	104.91	101.40	95.92	to	106.41	0.052
FEF$_{25-75}$ (% predicted)	73.40	56.35	to	92.05	81.70	67.24	to	97.29	0.089
PEFR (% predicted)	86.30	78.86	to	99.91	92.40	80.94	to	102.73	0.131
Bronchodilator response (spirometry)									
△FEV1 (%)	4.60	2.62	to	7.68	1.20	-1.72	to	4.22	<0.001
△FEV$_{25-75}$ (%)	16.00	7.89	to	29.76	10.20	2.02	to	20.91	0.065
IOS metrics parameters, baseline									
Zrs (kPa L^{-1} s)	0.90	0.75	to	1.05	0.70	0.62	to	0.89	<0.001
R5 (kPa L^{-1} s)	0.80	0.71	to	0.99	0.70	0.59	to	0.86	<0.001
R5-R20 (kPa L^{-1} s)	0.20	0.14	to	0.29	0.20	0.10	to	0.25	0.064
X5 (kPa L^{-1} s)	-0.20	-0.29	to	-0.15	-0.20	-0.24	to	-0.14	0.017
Ax (kPa/L)	2.00	1.09	to	2.86	1.30	0.73	to	2.25	0.014
Fres. ($^{-1}$ s)	21.20	17.60	to	25.50	19.20	15.82	to	22.56	<0.001
Bronchodilator response (IOS)									
△R5 (%)	20.00	14.46	to	31.90	10.00	6.71	to	21.80	<0.001
△R5-R20 (%)	50.00	28.53	to	63.86	43.11	6.77	to	49.25	0.008
△AX (%)	50.00	36.96	to	65.13	30.00	14.37	to	51.58	0.003
FENO (ppb)	27.80	16.25	to tot	54.00	38.50	23.00	to	56.50	0.345

ICS, inhaled corticosteroid; IOS, impulse oscillometry; Fres, resonant frequency; R5, resistance at 5 Hz; R20, respiratory resistance at 20 Hz; X5, respiratory reactance at 5 Hz; AX, area of reactance; Δ, percentage of bronchodilator response: IQR: interquartile range represents the distance between the 25th percentile and 75th percentile. P-value by Mann-Whitney U Test.
