# Supplementary material for: Small Airway Dysfunction Measured by Impulse Oscillometry and Fractional Exhaled Nitric Oxide Is Associated With Asthma Control in Children
Source: Front Pediatr. 2022 Jun 17;10:877681. doi: 10.3389/fped.2022.877681 (PMC9247317; doi:10.3389/fped.2022.877681)
Supplement: Supplementary file 3 [file Table_2.pdf]

Supplementary 2. Normal IOS cut-off values defined R5, R5-R20, Ax, Zrs, Fres > 95th percentile or X5 values <5th percentile based on healthy controls, and spirometry criteria for asthma with FEV1>80%, MMEF>60%, BDR<12%.

	Healthy control (n=140)		BDR < 12% (n=674)		FEV1 > 80% (n=623)		MMEF > 60% (n=598)	
	5th Percentile	95th Percentile	5th Percentile	95th Percentile	5th Percentile	95th Percentile	5th Percentile	95th Percentile
R5 (kPa L^{-1} s)	0.42	**0.89**	0.45	1.08	0.44	1.08	0.44	1.06
R5-R20 (kPa L^{-1} s)	0.00	**0.29**	0.02	0.40	0.02	0.38	0.02	0.37
X5 (kPa L^{-1} s)	**-0.26**	-0.07	-0.34	-0.08	-0.33	-0.08	-0.33	-0.08
Ax (kPa/L)	0.24	**2.30**	0.28	3.83	0.27	3.67	0.27	3.59
Zrs (kPa L^{-1} s)	0.44	**0.93**	0.47	1.14	0.46	1.12	0.45	1.11
Fres. ($^{-1}$ s)	9.86	**23.88**	10.53	26.97	10.57	26.97	10.33	26.05

Abbreviations: IOS, impulse oscillometry; Fres, resonant frequency; R5, resistance at 5 Hz; R20, respiratory resistance at 20 Hz; X5, respiratory reactance at 5 Hz; AX, area of reactance; Zrs, impedance; BDR, bronchodilator response of △FEV1.
