# Supplementary material for: Small Airway Dysfunction Measured by Impulse Oscillometry and Fractional Exhaled Nitric Oxide Is Associated With Asthma Control in Children
Source: Front Pediatr. 2022 Jun 17;10:877681. doi: 10.3389/fped.2022.877681 (PMC9247317; doi:10.3389/fped.2022.877681)
Supplement: Supplementary file 4 [file Table_3.pdf]

Supplementary 3. Predictive values of FENO, IOS and spirometry measurements in predicting asthma diagnosis.

		Cut-off	Criterion values and coordinates of ROC curve						Area under the ROC curve			
Variable		Value	Sensitivity	Specificity	PPV	NPV	LR+	LR-	AUC	SE	95% CI	P-value
FENO (ppb)	>=	20.00	0.807	0.850	0.956	0.524	5.391	0.227	0.886	0.012	0.861 - 0.910	<0.001
Zrs (kPa L^{-1} s)	>=	0.93	0.227	0.943	0.941	0.234	3.969	0.820	0.659	0.024	0.613 - 0.706	<0.001
R5 (kPa L^{-1} s)	>=	0.89	0.234	0.950	0.949	0.237	4.679	0.806	0.668	0.024	0.622 - 0.714	<0.001
R5-R20 (kPa L^{-1} s)	>=	0.29	0.186	0.950	0.937	0.226	3.714	0.857	0.700	0.025	0.651 - 0.748	<0.001
X5 (kPa L^{-1} s)	<=	-0.26	0.205	0.950	0.943	0.230	4.107	0.836	0.610	0.025	0.562 - 0.659	<0.001
Ax (kPa/L)	>=	2.30	0.257	0.950	0.954	0.242	5.143	0.782	0.687	0.024	0.640 - 0.734	<0.001
Fres. ($^{-1}$ s)	>=	23.88	0.166	0.950	0.930	0.222	3.321	0.878	0.668	0.025	0.618 - 0.717	<0.001
△R5 (%)	>=	40.0	0.032	1.000	1.000	0.205	–	0.968	0.612	0.026	0.561 - 0.663	<0.001
△R5-R20 (%)	>=	165.0	0.007	0.979	0.571	0.198	0.333	1.015	0.572	0.028	0.518 - 0.627	0.008
△AX (%)	>=	99.0	0.002	1.000	1.000	0.200	–	0.998	0.612	0.025	0.562 - 0.661	<0.001
FEV1 (% predicted)	<=	80.00	0.132	0.979	0.961	0.220	6.178	0.887	0.590	0.026	0.539 - 0.640	0.001
FVC (% predicted)	<=	80.00	0.125	0.929	0.875	0.210	1.753	0.942	0.540	0.027	0.486 - 0.593	0.146
FEV1/FVC (%)	<=	80.00	0.021	0.993	0.923	0.202	3.005	0.986	0.579	0.027	0.525 - 0.633	0.004
FEF$_{25-75}$ (% predicted)	<=	60.00	0.170	0.950	0.931	0.222	3.399	0.874	0.591	0.026	0.540 - 0.642	0.001
PEFR (% predicted)	<=	80.00	0.241	0.886	0.894	0.226	2.109	0.857	0.570	0.026	0.519 - 0.621	0.010
△FEV1 (%)	>=	12.00	0.038	0.964	0.805	0.200	1.035	0.999	0.537	0.026	0.486 - 0.588	0.179
△FEV$_{25-75}$ (%)	>=	30.00	0.150	0.870	0.821	0.204	1.150	0.978	0.512	0.026	0.460 - 0.564	0.665

PPV: positive predictive value; NPV: negative predictive value; LR+: positive likelihood ratio; LR−: negative likelihood ratio; AUC: Area under the curve.
