# Supplementary material for: Small Airway Dysfunction Measured by Impulse Oscillometry and Fractional Exhaled Nitric Oxide Is Associated With Asthma Control in Children
Source: Front Pediatr. 2022 Jun 17;10:877681. doi: 10.3389/fped.2022.877681 (PMC9247317; doi:10.3389/fped.2022.877681)
Supplement: Supplementary file 5 [file Table_4.pdf]

Supplementary 4. Predictive values for uncontrolled asthma among asthmatic children between FENO, IOS and spirometry measurements.

		Cut-off	Criterion values and coordinates of ROC curve						Area under the ROC curve			
Variable		Value	Sensitivity	Specificity	PPV	NPV	LR+	LR-	AUC	SE	95% CI	P-value
FENO (ppb)	>=	20.00	0.978	0.209	0.097	0.991	1.236	0.106	0.714	0.040	0.636 - 0.792	<0.001
Zrs (kPa L^{-1} s)	>=	0.93	0.333	0.781	0.118	0.931	1.524	0.853	0.617	0.044	0.531 - 0.702	0.010
R5 (kPa L^{-1} s)	>=	0.89	0.378	0.777	0.129	0.935	1.697	0.800	0.616	0.044	0.530 - 0.702	0.010
R5-R20 (kPa L^{-1} s)	>=	0.29	0.244	0.817	0.105	0.925	1.336	0.925	0.625	0.043	0.540 - 0.711	0.005
X5 (kPa L^{-1} s)	<=	-0.26	0.378	0.809	0.148	0.937	1.981	0.769	0.609	0.046	0.518 - 0.700	0.015
Ax (kPa/L)	>=	2.30	0.257	0.950	0.242	0.954	5.143	0.782	0.622	0.048	0.528 - 0.716	0.007
Fres. ($^{-1}$ s)	>=	23.88	0.333	0.847	0.160	0.936	2.177	0.787	0.606	0.047	0.514 - 0.698	0.018
△R5 (%)	>=	40.00	0.044	0.974	0.131	0.921	1.720	0.981	0.625	0.045	0.536 - 0.714	0.006
△R5-R20 (%)	>=	165.00	0.022	0.994	0.246	0.921	3.726	0.984	0.528	0.047	0.436 - 0.620	0.530
△AX (%)	>=	99.00	0.000	0.998	0.000	0.919	0.000	1.002	0.528	0.046	0.439 - 0.618	0.530
FEV1 (% predicted)	<=	80.00	0.489	0.899	0.297	0.953	4.832	0.569	0.734	0.044	0.647 - 0.820	<0.001
FVC (% predicted)	<=	80.00	0.333	0.893	0.214	0.939	3.115	0.747	0.640	0.048	0.546 - 0.734	0.002
FEV1/FVC (%)	<=	80.00	0.111	0.986	0.416	0.927	8.159	0.901	0.667	0.046	0.576 - 0.758	<0.001
FEF_{25-75} (% predicted)	<=	60.00	0.556	0.864	0.263	0.957	4.079	0.515	0.754	0.043	0.669 - 0.838	<0.001
PEFR (% predicted)	<=	80.00	0.467	0.780	0.156	0.944	2.123	0.684	0.686	0.044	0.599 - 0.772	<0.001
△FEV1 (%)	>=	12.00	0.267	0.982	0.566	0.939	14.904	0.747	0.785	0.039	0.709 - 0.861	<0.001
△FEV_{25-75} (%)	>=	30.00	0.422	0.871	0.222	0.945	3.267	0.664	0.732	0.040	0.654 - 0.810	<0.001

PPV: positive predictive value; NPV: negative predictive value; LR+: positive likelihood ratio; LR−: negative likelihood ratio; AUC: Area under the curve.
