# Supplementary material for: Small Airway Dysfunction Measured by Impulse Oscillometry and Fractional Exhaled Nitric Oxide Is Associated With Asthma Control in Children
Source: Front Pediatr. 2022 Jun 17;10:877681. doi: 10.3389/fped.2022.877681 (PMC9247317; doi:10.3389/fped.2022.877681)
Supplement: Supplementary file 6 [file Table_5.pdf]

Supplementary 5. Predictive values of FENO (>20 ppb) combined with IOS and spirometry measurements in predicting uncontrolled asthma among children.

Variable		Cut-off Value	Criterion values and coordinates of ROC curve Sensitivity	Specificity	PPV	NPV	LR+	LR-	Area under the ROC curve AUC	SE	95% CI	P-value
FENO>20 ppb combined												
Zrs (kPa L^{-1} s)	>=	0.93	0.356	0.878	0.203	0.940	2.907	0.734	0.722	0.037	0.650 - 0.795	<0.001
R5 (kPa L^{-1} s)	>=	0.89	0.356	0.876	0.200	0.940	2.861	0.736	0.721	0.037	0.649 - 0.794	<0.001
R5-R20 (kPa L^{-1} s)	>=	0.29	0.244	0.889	0.162	0.931	2.209	0.850	0.718	0.036	0.647 - 0.788	<0.001
X5 (kPa L^{-1} s)	<=	-0.26	0.356	0.880	0.205	0.940	2.953	0.733	0.709	0.039	0.633 - 0.784	<0.001
Ax (kPa/L)	>=	2.30	0.444	0.854	0.211	0.946	3.052	0.650	0.716	0.039	0.639 - 0.793	<0.001
Fres. ($^{-1}$ s)	>=	23.88	0.311	0.911	0.233	0.938	3.483	0.756	0.702	0.039	0.626 - 0.777	<0.001
△R5 (%)	>=	40.00	0.044	0.988	0.250	0.922	3.815	0.967	0.720	0.038	0.647 - 0.794	<0.001
△R5-R20 (%)	>=	165.00	0.022	0.998	0.500	0.921	11.444	0.980	0.647	0.039	0.570 - 0.724	0.001
△AX (%)	>=	99.00	0.000	1.000	–	0.920	–	1.000	0.650	0.039	0.574 - 0.725	0.001
FEV1 (% predicted)	<=	80.00	0.467	0.926	0.356	0.952	6.325	0.576	0.779	0.036	0.708 - 0.850	<0.001
FVC (% predicted)	<=	80.00	0.333	0.915	0.254	0.940	3.902	0.729	0.699	0.040	0.621 - 0.777	<0.001
FEV1/FVC (%)	<=	80.00	0.111	0.990	0.500	0.927	11.444	0.898	0.731	0.038	0.656 - 0.805	<0.001
FEF_{25-75} (% predicted)	<=	60.00	0.533	0.903	0.324	0.957	5.493	0.517	0.800	0.035	0.731 - 0.870	<0.001
PEFR (% predicted)	<=	80.00	0.444	0.847	0.202	0.946	2.897	0.656	0.750	0.037	0.677 - 0.823	<0.001
△FEV1 (%)	>=	12.00	0.267	0.988	0.667	0.939	22.889	0.742	0.818	0.034	0.752 - 0.884	<0.001
△FEV_{25-75} (%)	>=	30.00	0.422	0.911	0.292	0.947	4.727	0.634	0.788	0.035	0.719 - 0.856	<0.001

PPV: positive predictive value; NPV: negative predictive value; LR+: positive likelihood ratio; LR−: negative likelihood ratio; AUC: Area under the curve.curve.
